# *Legionella pneumophila* infection presenting as headache, confusion and dysarthria in a human immunodeficiency virus-1 (HIV-1) positive patient: case report

**DOI:** 10.1186/1471-2334-12-225

**Published:** 2012-09-22

**Authors:** Nathaniel M Robbins, Aditi Kumar, Barbra M Blair

**Affiliations:** 1 Department of Neurology, University of California, 505 Parnassus Ave. M-798, San Francisco, CA, 94137, USA; 2 Department of Medicine, Mount Auburn Hospital, 330 Mount Auburn St., Cambridge, MA, 02138, USA; 3 Harvard Medical School, Boston, MA, USA

**Keywords:** Legionella, Legionellosis, Splenium, Corpus callosum, MRI, HIV, Encephalopathy

## Abstract

**Background:**

*Legionella pneumophila* is a common cause of community-acquired pneumonia. Central nervous system dysfunction is common, and diagnosis in the absence of pulmonary symptoms can be challenging. Here we describe an atypical clinical presentation of *Legionella* infection in a patient with HIV who was found to have an unusual neuroradiologic lesion that further served to obscure the diagnosis. This is the first such description in a patient with Legionellosis and HIV coinfection.

**Case presentation:**

A 43 year-old HIV positive man presented to our hospital with dysarthria, fevers, headache, and altered mental status. Initial work-up revealed pneumonia and a lesion of the splenium of the corpus callosum on magnetic resonance imaging. He was subsequently diagnosed with *Legionella* pneumonia and treated with complete symptom resolution.

**Conclusions:**

Neurologic abnormalities are frequent in Legionellosis, but the diagnosis may be difficult in the absence of overt respiratory symptoms and in the presence of HIV coinfection. A high index of suspicion and early initiation of empiric antibiotics is imperative since early treatment may help prevent long-term sequelae. Neuroimaging abnormalities, though rare, can help the physician narrow down the diagnosis and avoid unnecessary invasive testing. Future studies should aim to elucidate the as yet unknown role of neuroimaging in the diagnoses and prognostication of Legionellosis, as well as the interaction between *Legionella* infection and HIV.

## Background

*Legionella pneumophila* is now recognized as a major cause of both community-acquired pneumonia [1] and nosocomial pneumonia [2], accounting for between 2 and 15% of pneumonias requiring hospitalization [3]. The central nervous system (CNS) is involved in up to 50% of patients [4], and permanent sequalae are among the most feared complications of infection. While relatively straightforward to treat, diagnosis of Legionellosis can be obscured by multiorgan system involvement, particularly in the absence of obvious respiratory symptomatology. There is rarely evidence of CNS infection by neuroimaging, cerebrospinal fluid (CSF) analysis, or pathologic evaluation [5-8], which further confuses diagnosis in atypical cases. In the case study reported below, we describe the challenge of diagnosing *Legionella* in a patient with HIV who presented with neurologic deficits and a hyperintense lesion of the splenium of the corpus callosum (SCC) on magnetic resonance imaging (MRI). To the best of our knowledge, this is the first such description of these radiologic findings in a patient with Legionellosis and HIV coinfection. This report highlights the pitfalls, challenges, and importance of diagnosing *Legionella* infection when neurologic symptoms predominate.

## Case presentation

### Initial presentation

A 43 year-old male was referred to the Emergency Department (ED) of our hospital after his workplace colleagues noted increasingly strange behavior for three days. The patient arrived complaining of generalized fatigue, lethargy, fevers, severe band-like headache, slurred speech, and increasing confusion. Though it was felt that the patient’s history was not completely reliable secondary to confusion, he was able to deny throat pain, chest pain, diarrhea, abdominal pain, recent sick contacts, or recent travel. Review of systems was limited secondary to altered mental status, but he did admit to mild dry cough.

Though initially denying immune compromise in the ED, the patient later disclosed he was HIV+. He could not recall his CD4 count but stated that his last viral load was undetectable. Past medical history was significant for hypertension and hypercholesterolemia. Outpatient medications included Atripla, spironolactone, and gemfibrozil. He had a history of unprotected anal sex with men but denied new partners. He had no recent contact with animals. He smoked a pack of cigarettes per day but did not drink or use drugs.

In the ED, the patient’s temperature was 103.9°F (39.9°C). Heart rate was 121 beats per minute, and respiratory rate was 30 breaths per minute. Oxygen saturation was 94% on room air. Blood pressure was 142/90. The patient appeared unwell. He was somnolent but easily aroused. Pertinent findings on general physical exam included a supple neck and decreased breath sounds diffusely. On neurologic exam, he was noted to be dysarthric. He had general slowness of thought and processing. He was slightly confused, though he was oriented and able to name the days of the week forwards and backwards if given sufficient time.

Initial laboratory evaluation was notable for a white blood cell (WBC) count of 10.1 × 10^3^ cells/mm^3^, [lab reference range 4.0–10.8], hemoglobin of 15.7 g/dL [lab range 14.0–18.0], platelet count of 135 × 10^3^/mm^3^ [lab range 150–350]. Automated differential showed 87.5% segmented cells (lab range 30-85%), 9.3% lymphocytes (lab range 15-55%), 2.4% monocytes (lab range 0-10%), 0.8% eosinophils (lab range 0-5%), and 0% basophils. No manual differential was performed. A basic metabolic panel showed serum sodium of 134 mmol/L [lab range 137–145], serum potassium of 4.2 mmol/L (lab range 3.5-5.1), serum chloride of 100 mmol/L (lab range 98–107), and CO_2_ of 19 mmol/L [lab range 22.0–30.0]. Blood urea nitrogen, serum creatinine, and serum glucose were elevated at 33 mg/dL [lab range 9–20], 1.8 mg/dL [lab range 0.7–1.3] and 159 mg/dL [lab range 70–99], respectively. C-reactive protein (CRP) was high at 581 mg/dL [lab range 0–10]. Liver function studies showed an albumin level of 4.4 g/dL (lab range 3.5-5.0), total protein of 7.6 g/dL (lab range 6.3-8.2), total bilirubin of 0.7 mg/dL (lab range 0.2-1.3), direct bilirubin of 0.5 mg/dL (lab range 0–0.3), alkaline phosphatase of 62 U/L (lab range 38–126), alanine aminotransferase (ALT) of 49 U/L (lab range 13–69), and aspartate aminotransferase (AST) of 89 U/L (lab range 15–46). Urinalysis showed 2+ albumin, 3+ blood, trace WBC esterase, negative nitrite, 5–10 WBC, 0–5 red blood cells (RBC), 5–10 epithelial cells, 5–10 hyaline casts, 3+ amorphous cells, and 1+ mucous. A non-contrast computed tomography (CT) scan of the head did not reveal any abnormalities. Nasopharyngeal influenza assay was negative.

Due to the combination of altered mental status, headache, and fever, a lumbar puncture was performed. The results demonstrated an elevated cerebrospinal fluid (CSF) glucose of 97 mg/dL [normal 40–70], CSF protein of 37 mg/dL [normal 12–60], and normal cell count and gram stain. Blood cultures were sent.

**Figure 1:**
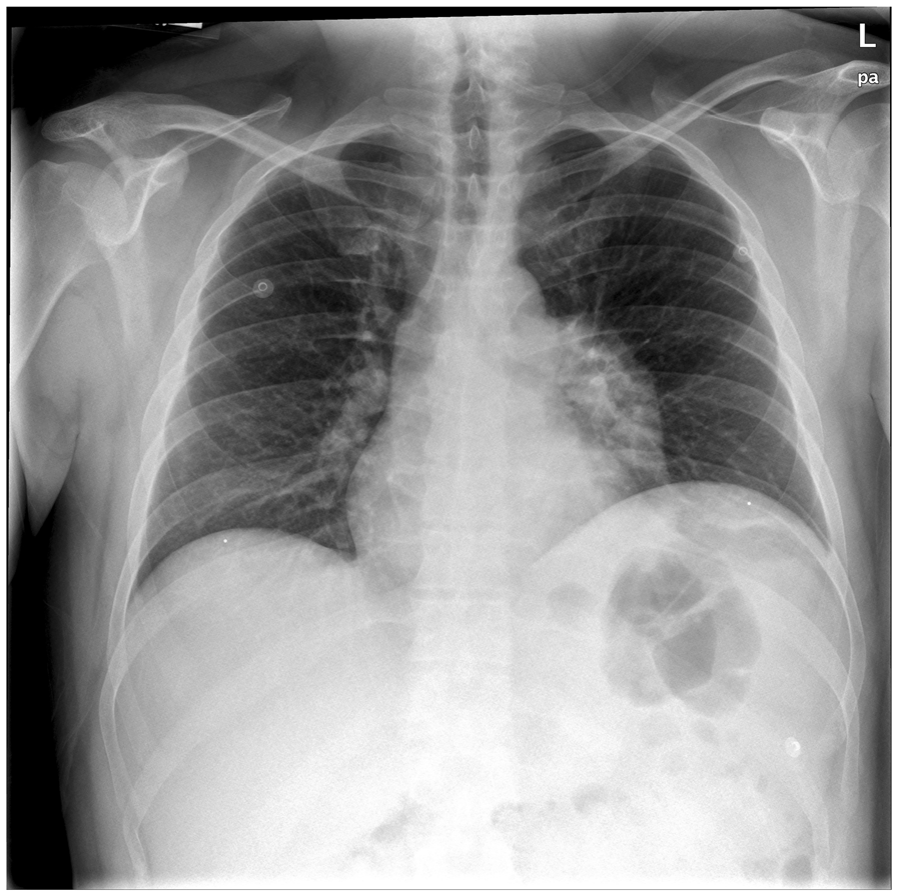
Chest x-ray demonstrating a left hilar mass.

**Figure 2:**
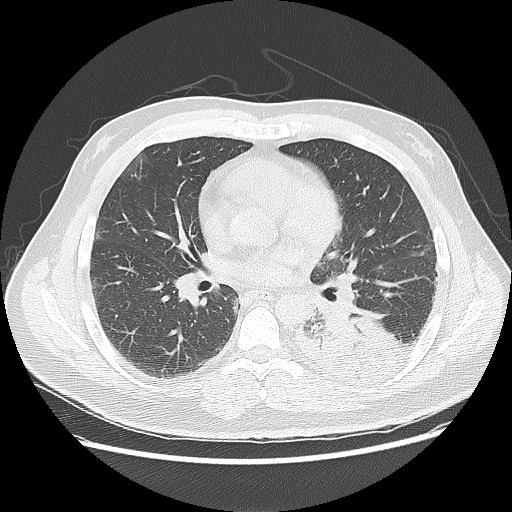
Chest CT demonstrating left-sided pneumonia.

A chest x-ray was done and was read as a left hilar mass extending down towards the left lower lobe (see Figure 1). A chest CT was obtained to better characterize the hilar mass (see Figure 2*).* This chest CT demonstrated a left lower lobe consolidation with air bronchograms suggesting a diagnosis of pneumonia. Based upon the aforementioned findings, the patient was started on azithromycin and ceftriaxone for community-acquired pneumonia, as well as empiric acyclovir for herpes encephalitis.

**Figure 3:**
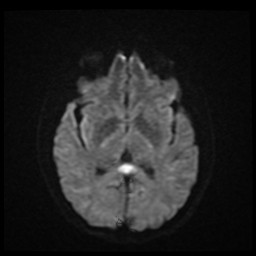
Axial MRI brain without contrast demonstrating restricted diffusion of the SCC on DWI.

**Figure 4:**
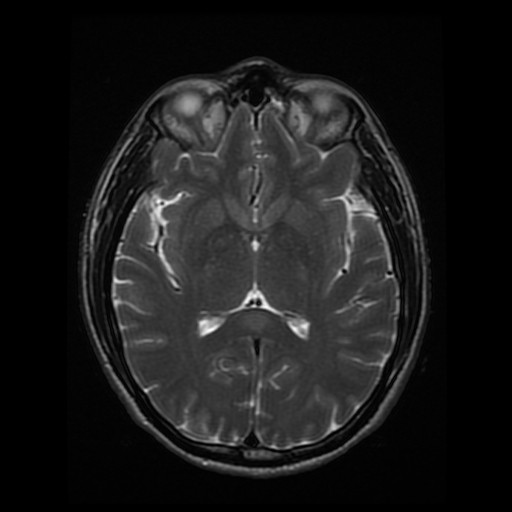
Axial T2-weighted MRI without contrast demonstrating focal hyperintensity of the SCC.

Given the findings of dysarthria and altered mental status in an HIV + individual with unknown CD4+ count, a brain MRI was obtained. No contrast was administered secondary to acute renal injury. The diffusion-weighted coronal cut can be seen in Figure 3, demonstrating abnormal restricted diffusion and swelling of the splenium of the corpus callosum without other significant abnormalities. Abnormal T2-weighted signal in the SCC can be seen in Figure 4*.* These MRI findings in an HIV + individual raised concern for CNS opportunistic infection. However, since the patient was hemodynamically stable, was on antiretroviral therapy, and had a negative lumbar puncture, it was decided to treat the pneumonia initially and closely monitor the patient’s mental status.

### Hospital course

Over the first two to three days of hospitalization, the patient continued to have high fevers, confusion, and dysarthria. His laboratory markers improved on antibiotics. His serum CRP trended down from 581 mg/L at admission to 200 mg/L on day three. After day three the patient began to demonstrate significant clinical improvement. The patient’s admission CD4+ count returned on day three at 156 cells/mm^3^, and an HIV viral load came back on day four at <20 copies/mL. On day four, urine antigens for *Streptococcus pneumoniae* and *Legionella pneumophila* serogroup 1 were sent. By day five the patient’s neurologic symptoms had resolved, as had his acute renal insufficiency. This same day his *Legionella pneumophila* urinary antigen returned positive.

On hospital day six a repeat MRI was obtained to monitor radiologic progression of the SCC lesion. This repeat study showed partial interval clearing of the previously noted abnormalities: restricted diffusion, abnormal increased flair, and abnormal T2 signally in the SCC. On hospital day seven the patient was discharged home on oral azithromycin. He had no residual deficits. Initial CSF viral studies including herpes simplex virus 1 and 2, varicella, cytomegalovirus, enterovirus, and JC virus all returned negative, as did the syphilis, hepatitis B, and hepatitis C serologies. Sputum for acid fast bacilli and routine culture were negative. Legionella antibody IgG titers sent on day four later came back at 1:64.

## Discussion

### *Legionella* and the CNS

Here we present an HIV + patient who presented with confusion, dysarthria, and fever, and was subsequently found with a lesion of the SCC on MRI before being diagnosed with *Legionella* pneumonia. “Legionnaire’s disease” was first described in the late 1970s in a series of articles outlining an emerging respiratory epidemic [9-14]. While relatively straightforward to treat, the challenge in Legionellosis is to make a diagnosis early and to initiate appropriate treatment, as delaying therapy is associated with increased mortality [15]. *Legionella* is effectively treated with macrolides, quinolones, or tetracyclines [1].

CNS involvement is a feared and relatively common sequela of Legionellosis, as has been described in detail [7,16-23]. Neurologic problems range from peripheral neuropathy [24] to myositis [25] to isolated nerve palsies [26] to acute disseminated encephalomyelitis (ADEM) [27-29]. Altered mental status and headache were the most frequent neurologic symptoms in one series [7], occurring in 29.6% and 28.7% of patients, respectively. Cerebellar signs are less common in Legionellosis, but also well-described [6,30]. As mentioned previously, it is rare to observe evidence of infection by neuroimaging, cerebrospinal fluid (CSF) analysis, or pathologic evaluation [5-8]. Electroencephalogram (EEG) may show diffuse slowing or may be normal [6]. In severe cases with neurologic involvement, focal deficits may not fully resolve even with adequate antibiotic coverage [6,7]. Fortunately this patient was treated upon presentation with adequate empiric antibiotics and made a complete recovery.

One prominent feature of this case was the unusual radiologic findings that accompanied the patient’s encephalopathy. Though radiologic or pathologic confirmation of Legionellosis is reported to be rare, one previously recorded case study on Legionellosis with cerebellar involvement also describes an isolated enhancing lesion of the SCC on MRI [4]. This nonspecific reversible lesion [31] has been described previously in a host of infectious [32-35] and non-infectious [34] causes, including antiepileptic drug-withdrawal, high-altitude cerebral edema, and metabolic disorders [35]. The frequency of this lesion is likely underreported, since most patients who present with altered mental status do not receive an MRI. The cellular pathophysiologic cause of an isolated SCC is as yet unknown, though various theories have been posited [34,35]. In the neuropsychiatric and neuroanatomical literature, the SCC is thought to mediate interhemispheric transfer of information, but its precise role in human cognition remains to be defined [36]. We cannot state with certainty what role the SCC abnormality played in the presentation of our patient, as the causes and correlates of this abnormality are not well-studied to this point.

Splenial lesions on MRI, if symptomatic, are most commonly associated with delirium, ataxia, recent seizure, hemispheric disconnection syndrome, dysarthria, increased tone, or headache. These nonspecific findings do not easily localize anatomically [34]. Because of this apparent contradiction, Morgan et al. postulated that additional areas of the brain may be affected even if MRI cannot detect involvement [4]. Working on this hypothesis, Imai and colleagues found evidence of diffuse cerebellar and frontal lobe hypoperfusion on single photon emission CT in a patient with *Legionella* pneumonia who presented similarly to the patient described in the case study: respiratory symptoms, confusion, dysarthria, and an isolated hyperintense reversible SCC lesion on MRI diffusion-weighted imaging (DWI) [37]. It remains to be seen what significance these radiologic findings hold for the diagnosis and treatment of the *Legionella* or the other entities listed above, but clearly more research is needed in order to understand the utility of this radiologic abnormality in diagnosing and prognosticating encephalopathy.

### *Legionella* and HIV

Coinfection with HIV confused timely diagnosis in this patient with Legionellosis. *Legionella* pneumonia has been observed but not well-studied in HIV + individuals [38,39], and it is unknown if HIV infection predisposes an individual to more severe Legionellosis or CNS involvement. This patient’s CD4+ count was reduced, but Legionellosis is known to cause lymphopenia in the acute setting [40], so the role of HIV-induced lymphopenia as a risk-factor for *Legionella* infection in this context is unclear. At the least, HIV seropositivity and the predominance of neurologic symptomatology in this case distracted the clinicians from making a prompt diagnosis, highlighting the importance of early empiric antibiotic therapy.

## Conclusions

We describe an unusual case of *Legionella* pneumonia in a patient with HIV infection who presented with altered mental status, dysarthria, and neuroimaging abnormalities that cleared with clinical improvement. In this case, appropriate antibiotic therapy was started immediately upon presentation, even though the diagnosis of Legionellosis was not made until urinary antigens came back four days later. The patient did indeed make a complete recovery, and we presume early antibiotic therapy helped in this convalescence to some extent.

This patient underwent brain MRI because he presented acutely ill with confusion and dysarthria in the setting of HIV infection without an obvious diagnosis. MRI revealed an isolated defect of the SCC. The significance of this lesion and the utility of advanced imaging techniques in encephalopathy in general and Legionellosis specifically is not known, since to date no prospective studies have evaluated the utility of either MRI or SPECT in the work-up of altered mental status or other neurologic signs in Legionellosis. Though cost is currently a significant barrier, these imaging modalities may be useful to assess prognosis, determine optimal treatment length, and possibly even diagnose Legionellosis when CNS symptoms predominate. Further research in this area is required. Regardless, clinicians need to be aware of this nonspecific finding so as to avoid unnecessary invasive procedures in the setting of encephalopathy.

The other challenging aspect of this patient’s presentation was his coinfection with HIV. Given the limited research into the relationship between *Legionella* and HIV, additional investigation is warranted in order to better understand the immunologic interplay.

In summary, clinicians should consider *Legionella* infection in patients who present with altered mental status or neurologic symptoms, even when few respiratory symptoms are reported. Failure to send *Legionella* urinary antigens on presentation in this case led to a delay in diagnosis, though fortunately this patient was initially placed on adequate empiric therapy anyway. In the absence of overt respiratory symptoms, clinicians need to maintain a high index of suspicion as early therapy may help prevent permanent neurologic sequelae.

## Consent

Written informed consent was obtained from the patient for publication of this Case Report and any accompanying images. A copy of the written consent is available for review by the Series Editor of this journal.

## Abbreviations

ADEM: Acute disseminated encephalomyelitis; ALT: Alanine aminotransferase; AST: Aspartate aminotransferase; CD4: Cluster of differentiation; CNS: Central nervous system; CRP: C-reactive protein; CSF: Cerebrospinal fluid; CT: Computed tomography; DWI: Diffusion-weighted imaging; ED: Emergency department; EEG: Electroencephalogram; HIV: Human immunodefiency virus; IgG: Immunoglobulin G; MRI: Magnetic resonance imaging; SCC: Splenium of the corpus callosum; SPECT: Single photon emission CT.

## Competing interests

The authors declare that they have no competing interests.

## Authors’ contributions

All authors were involved in the clinical care of the patient. All authors participated equally in writing and editing this manuscript. All authors read and approved the final manuscript.

## Authors’ information

NMR is a Resident in Neurology at the University of California at San Francisco. AK is a Resident in Internal Medicine at Mount Auburn Hospital, a teaching hospital of Harvard University, and a Clinical Fellow in Medicine at Harvard Medical School. BMB is an Instructor in Medicine in the Division of Infectious Disease at Mount Auburn Hospital and Harvard Medical School.

## Pre-publication history

The pre-publication history for this paper can be accessed here:

http://www.biomedcentral.com/1471-2334/12/225/prepub
